# Trends in mortality rates and correlations between intracranial injuries and external causes: A Japanese population study

**DOI:** 10.1371/journal.pone.0300846

**Published:** 2024-05-08

**Authors:** Ryo Shimada, Kazuhiko Kibayashi

**Affiliations:** Department of Forensic Medicine, School of Medicine, Tokyo Women’s Medical University, Tokyo, Japan; Duke University Medical Center: Duke University Hospital, UNITED STATES

## Abstract

The age-standardized incidence of head trauma in 2016 was 369 per 100,000 people worldwide. The Western Pacific region, including Japan, had the highest incidence. This study aimed to extract ICD-10 code data for intracranial injury (S06) and external causes of morbidity and mortality (V01–Y89), analyze their characteristics and interrelationships, and contribute to these diseases’ prevention, treatment, and prognosis. The number of deaths according to injury type and external cause type of intracranial injury published by the Japanese government was statistically analyzed using JoinPoint, and univariate distribution and multivariate correlation were conducted using JMP Software. From 1999–2021, there was a downward trend in the number of deaths because of intracranial injuries: mortality from intracranial injuries was higher among those aged ≥65 years. Conversely, mortality from intracranial injuries was lower among those aged ≤14 years. Among deaths from intracranial injury, mortality from diffuse brain injury and traumatic subdural hemorrhage was more common. Among deaths from external causes of intracranial injury, mortality from falls, transport accidents, and other unforeseen accidents was more common. Mortality because of intracranial injuries increased significantly during the 2011 Great East Japan Earthquake. For some age groups and sexes, there were significant inverse correlations of mortality with traumatic subdural hemorrhage and traumatic subarachnoid hemorrhage for transport accidents, intentional self-harm and assault, and diffuse brain injury and focal brain injury for falls. We believe that the data presented in this study will be useful for preventing and treating intracranial injuries and for developing administrative measures to reduce intracranial injuries.

## Introduction

In 2016, the global average age-standardized incidence of traumatic brain injury (TBI) was 369 (331–412) per 100,000 individuals. According to the Global Burden of Disease Study, the highest rate was observed in the Western Pacific region at 483 (419–554) per 100,000 people, followed by the Southeast Asia region at 454 (395–518) per 100,000 people. The lowest rate was observed in Africa at 280 (254–308) per 100,000 people, followed by the Eastern Mediterranean region at 297 (256–342) per 100,000 people. Japan falls under the Western Pacific region [1].

TBI causes a significant number of deaths and disabilities worldwide [2, 3]. The mortality rate after severe TBI can be as high as 50%, and its reduction is a major public health challenge [4–6]. From 1998–2001, 525 of 1,002 TBIs in Japan resulted in death, with a mortality rate of 52.4%, according to the final report of the Japan Neurotrauma Data Bank Project [7].

This study aimed to extract ICD-10 code data for intracranial injury (S06) and external causes of morbidity and mortality (V01–Y89) in Japan, analyze their characteristics and interrelationships, and contribute to these diseases’ prevention, treatment, and prognosis.

## Material and methods

We performed a statistical analysis by extracting the number of intracranial injury deaths from each type of intracranial injury, each type of external cause, and the population from 1999–2021, published by the Japanese government. We calculated the mortality rate per 100,000 people.

We analyzed and compared the main categories of causes of mortality: intracranial injury (S06), diffuse brain injury (S06.2), focal brain injury (S06.3), epidural hemorrhage (S06.4), traumatic subdural hemorrhage (S06.5) and traumatic subarachnoid hemorrhage (S06.6) for intracranial injuries by injury type. We also examined external causes of morbidity and mortality (V01–Y89), transport accidents (V01–V99), falls (W00–W19), intentional self-harm (X60–X84), assault (X85–Y09), and other unforeseen accidents (remainder W00–X59 [8]. The numbers indicate the Japanese classification and summary codes) for intracranial injuries with an external cause.

The number of deaths due to each type of intracranial injury that occurred between 1999 and 2021 was extracted from “Table dead 1 (1) ICD-10 codes A to T, Number of deaths, by cause of death (basic classification of cause of death), sex, and age (5-year age group)” in the “Year” portion of the statistics relating to the “Cause of death” component of the “Demographic Survey of Vital Statistics” section published on the E-Stat Portal Site of the Official Statistics of Japan [9].

The number of deaths from intracranial injury due to external causes that occurred from 1999–2021 was extracted from “Table last volume 10: Number of deaths from external causes, by age (specific class), external cause (simple classification of cause of death), sex, and effect of external cause” in the “Year” portion of the statistics relating to the “Death” component of the “Demographic Survey of Vital Statistics” section published on the E-Stat Portal Site of the Official Statistics of Japan [10].

Population data for the 1999–2021 period was extracted from Population estimates/yearly/annual report/table Japan 2 or 3 “Population by Age (Five-Year Groups) and Sex, Monthly Estimates- Total Population, Japanese Population, the First Day, Each Month” in the “Yearly” portion of the statistics relating to the “Annual Report” component of the “Population Estimates” section published on the E-Stat Portal Site of the Official Statistics of Japan [11]. The standard population was the population of 2010 [12].

Statistical analyses were performed using JMP Pro Software version 16, released in 2021 by JMP Statistical Discovery LLC (SAS Institute Japan, Co. Ltd., Tokyo, Japan), and the JoinPoint Regression Software (National Cancer Institute). Differences were considered statistically significant at *p* ≤ 0.05. Confidence intervals (CI) were set at 95% and calculated using the univariate distribution platform of JMP. Spearman’s rank correlation of non-parametric statistical tests was used to compare the correlations of mortality between intracranial injuries due to injury type and external cause type using the multivariate correlation platform of JMP. We tested whether the apparent change in trend was statistically significant using JoinPoint Software. The number of fatalities, population, standard population, adjustment variable (age group), independent variable (year), and variable (sex) were entered, and age-adjusted rates were calculated using log transformation. The trend period, annual percentage change (APC) with a 95%CI, and average annual percentage change (AAPC) with a 95%CI were obtained [13].

## Results

### Intracranial injuries classified by injury type

Table 1 shows the average number of deaths and mortality rates by sex and age group because of intracranial injury (S06) by injury type from 1999–2021. The mortality rate for those aged ≥80 years was the highest for both sexes at 79.86 (95% CI, 79.81–79.90) per 100,000 for males and 28.18 (95% CI, 28.15–28.20) per 100,000 for females, followed by that for the 65–79-year age group at 19.68 (95% CI, 19.65–19.71) per 100,000 for males and 7.63 (95% CI, 7.60–7.66) per 100,000 for females; the 5–14-year age group had the lowest mortality rate for both sexes at 0.81 (95% CI, 0.79–0.83) per 100,000 for males and 0.49 (95% CI, 0.48–0.51) per 100,000 for females. All of the subdivisions of S06 had a high mortality rate in those aged ≥80 years, especially diffuse brain injury (S06.2) at 22.00 (95% CI, 21.91–22.09) per 100,000 for males and 6.71 (95% CI, 6.67–6.76) per 100,000 females and traumatic subdural hemorrhage (S06.5) with 47.17 (95% CI, 47.14–47.20) per 100,000 males and 17.86 (95%CI, 17.84–17.87) per 100,000 females. Mortality was lower in the younger age groups. It was lowest in the 5–14-year age group, especially for focal brain injury (S06.3) at 0.04 (95% CI, 0.03–0.05) per 100,000 in males and 0.04 (95% CI, 0.03–0.04) per 100,000 in females, and for epidural hemorrhage (S06.4) at 0.04 (95% CI, 0.03–0.05) per 100,000 in males and 0.02 (95% CI, 0.01–0.03) per 100,000 in females.

**Table 1:** Average number of deaths and mortality rates because of intracranial injury by injury type in 1999–2021 by sex and age group in Japan.

	Intracranial injury		Diffuse brain injury		Focal brain injury	
	(S06)		(S06.2)		(S06.3)	
Age (years)	Average number	Mortality rate (95% CI)	Average number	Mortality rate (95% CI)	Average number	Mortality rate (95% CI)
Male						
0–4	35	1.5 (1.46–1.54)	15	0.71 (0.68–0.74)	0.4	0.04 (0.03–0.06)
5–14	43	0.81 (0.79–0.83)	29.3	0.6 (0.58–0.62)	1.3	0.04 (0.03–0.05)
15–44	827.6	3.75 (3.73–3.77)	552.2	2.61 (2.60–2.63)	16.6	0.08 (0.07–0.08)
45–64	1118.1	6.67 (6.65–6.69)	573.3	3.67 (3.64–3.69)	20.5	0.12 (0.12–0.13)
65–79	1954.8	19.68 (19.65–19.71)	779	8.49 (8.45–8.53)	24.5	0.28 (0.27–0.29)
≥80	2333.2	79.86 (79.81–79.9)	612.7	22 (21.91–22.09)	14.6	0.53 (0.52–0.55)
Overall	6320.5	9.77 (9.77–9.78)	2566.8	4.18 (4.17–4.19)	78.2	0.13 (0.12–0.13)
Female						
0–4	22.7	0.99 (0.96–1.02)	11.1	0.53 (0.50–0.56)	0	0.04 (0.04–0.04)
5–14	23.1	0.49 (0.48–0.51)	15.6	0.36 (0.34–0.38)	0.6	0.04 (0.03–0.04)
15–44	277.5	1.26 (1.25–1.27)	183.3	0.86 (0.85–0.86)	4.8	0.03 (0.03–0.03)
45–64	363.3	2.19 (2.18–2.21)	190.3	1.23 (1.22–1.25)	5.9	0.04 (0.04–0.05)
65–79	845.9	7.63 (7.60–7.66)	323.5	3.32 (3.28–3.35)	11.8	0.12 (0.11–0.12)
≥80	1574.4	28.18 (28.15–28.2)	344.9	6.71 (6.67–6.76)	10.4	0.21 (0.20–0.22)
Overall	3108.8	4.57 (4.57–4.57)	1070.2	1.69 (1.69–1.7)	33.7	0.05 (0.05–0.05)
	Epidural hemorrhage		Traumatic subdural hemorrhage		Traumatic subarachnoid hemorrhage	
	(S06.4)		(S06.5)		(S06.6)	
Age (years)	Average number	Mortality rate (95% CI)	Average number	Mortality rate (95% CI)	Average number	Mortality rate (95% CI)
Male						
0–4	0.7	0.06 (0.05–0.07)	11.2	0.51 (0.48–0.54)	2.7	0.11 (0.10–0.12)
5–14	0.7	0.04 (0.03–0.05)	4	0.09 (0.08–0.1)	2.6	0.05 (0.05–0.06)
15–44	10.4	0.05 (0.05–0.05)	86.4	0.38 (0.37–0.38)	53	0.22 (0.21–0.22)
45–64	22.2	0.14 (0.14–0.14)	300.5	1.74 (1.73–1.74)	85.9	0.47 (0.47–0.48)
65–79	30.9	0.32 (0.32–0.33)	827.4	8.19 (8.17–8.2)	165.1	1.57 (1.56–1.58)
≥80	37.6	1.32 (1.31–1.34)	1378.1	47.17 (47.14–47.2)	205.8	6.48 (6.42–6.54)
Overall	102.5	0.16 (0.16–0.16)	2608.9	4.13 (4.12–4.13)	526.5	0.81 (0.80–0.81)
Female						
0–4	0.3	0.05 (0.03–0.06)	6.6	0.32 (0.30–0.34)	0.7	0.09 (0.07–0.11)
5–14	0.4	0.02 (0.01–0.03)	2.5	0.06 (0.05–0.07)	0.6	0.04 (0.03–0.05)
15–44	2.5	0.01 (0.01–0.02)	29.5	0.13 (0.13–0.14)	9.6	0.07 (0.07–0.07)
45–64	6.7	0.04 (0.04–0.05)	98.6	0.57 (0.56–0.57)	14.2	0.13 (0.12–0.13)
65–79	14.8	0.14 (0.13–0.14)	374	3.24 (3.23–3.25)	49.2	0.58 (0.57–0.59)
≥80	29.8	0.55 (0.54–0.56)	1002.1	17.86 (17.84–17.87)	96.8	2.21 (2.20–2.22)
Overall	54.6	0.08 (0.08–0.08)	1513.4	0.03 (0.03–0.03)	173.4	0.37 (0.37–0.37)

The mortality rate indicates the average number per 100,000 population. CI indicates the confidence interval.

**Fig 1:**
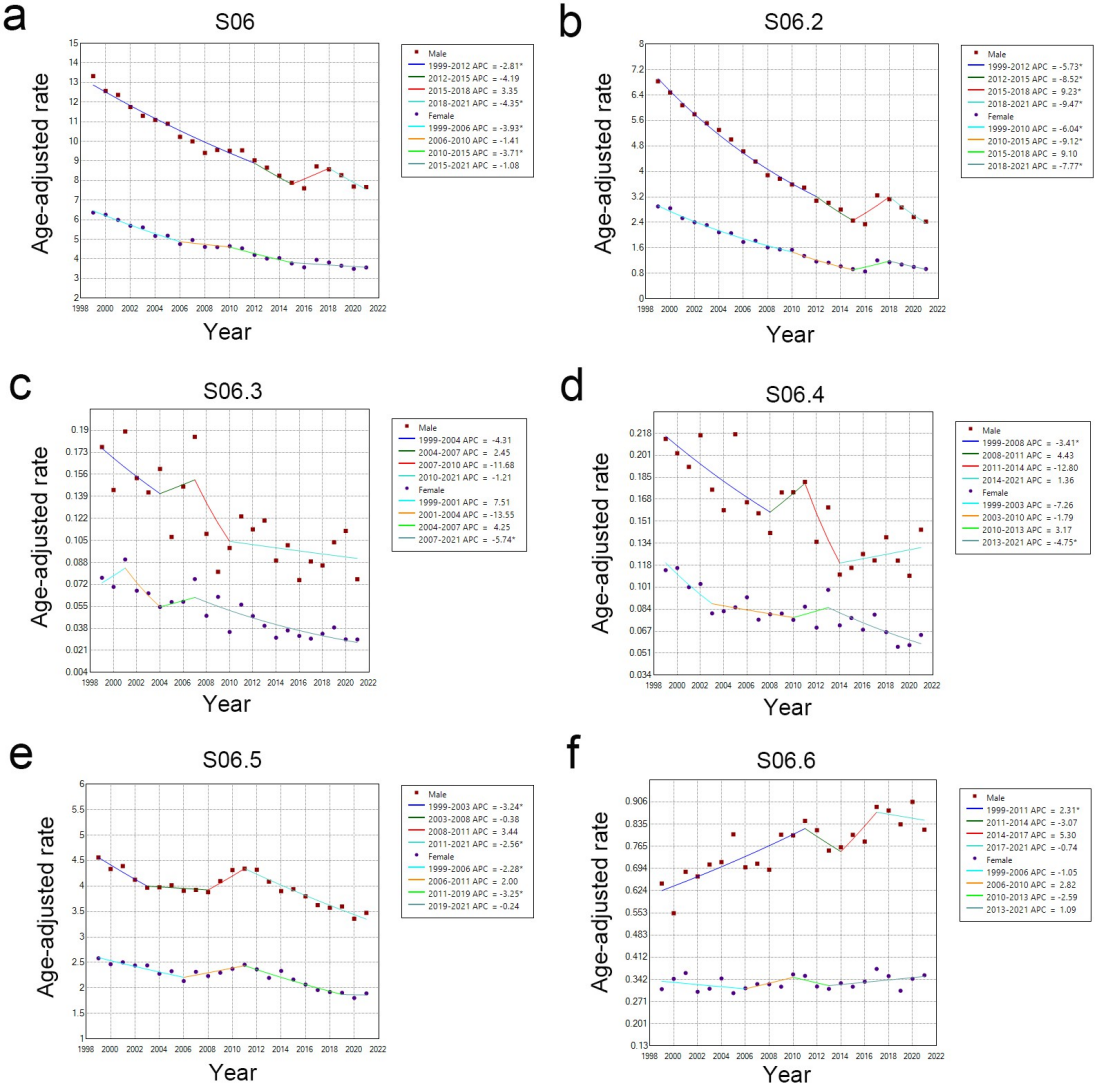
Trends in the age-adjusted rate of change of intracranial injury deaths by injury type from 1999–2021 in Japan: Analyzed using the JoinPoint regression program for males and females. APC: annual percentage of change. S06: intracranial injury, S06.2: diffuse brain injury, S06.3: focal brain injury, S06.4: epidural hemorrhage, S06.5: traumatic subdural hemorrhage, S06.6: traumatic subarachnoid hemorrhage.

We analyzed trends in the statistical rate of change in intracranial injury-related deaths by injury type from 1999–2021 using JoinPoint Regression Software. Both male and female age-adjusted rates showed a decreasing trend for S06, with a significant difference in the AAPC for both sexes. There was a significant difference in the APC for males from 1999–2012 and 2018–2021 and for females from 1999–2006 and 2010–2015 (Fig 1, S1 and S2 Tables). In the subdivisions of S06, the age-adjusted mortality rates from 1999–2021 showed a decreasing trend in S06.2, except for an increase in 2017, for both sexes; S06.3, except for an increase in 2007; and S06.4, except for an increase in 2011 for males and in 2013 for females; they remained almost unchanged for S06.5, except for an increase in 2011; and a slightly increasing trend for traumatic subarachnoid hemorrhage (S06.6) for both sexes, except for a decrease in 2013. There was a significant difference in AAPC for both sexes for S06.2 and S06.5. The *P*-values, indicating significant differences in all period trends for APCs in S06.2, were <0.05 for both sexes, except in the 2015–2018 period trend for females. There were significant differences in the trend period of APC for S06.5 in the 1999–2003 and 2011–2021 periods for males and the 1999–2006 and 2011–2019 periods for females. In addition, the APC trend periods with significant differences were the 1999–2008 period for S06.4, the 1999–2011 period for S06.6 for males, and the 2013–2021 period for S06.4 for females (Fig 1, S1 and S2 Tables).

### Intracranial injuries classified by external cause types

Table 2 shows the average number of deaths and mortality rates by sex and age group because of intracranial injury by external cause from 1999–2021. The mortality rate for external causes of morbidity and mortality (V01–Y89) for persons aged ≥80 years was the highest for both sexes at 79.86 (95% CI, 79.82–79.91) per 100,000 for males and 28.18 (95% CI, 28.15–28.21) per 100,000 for females, followed by the 65–79 age group for both sexes at 19.68 (95% CI, 19.65–19.72) per 100,000 for males and 7.63 (95% CI, 7.60–7.66) per 100,000 for females; the 5–14-year age group had the lowest rate for both sexes at 0.81 (95% CI, 0.80–0.83) per 100,000 for males and 0.49 (95% CI, 0.48–0.52) per 100,000 for females. These values were almost identical to intracranial injuries (S06) by injury type. All V01–Y89 subdivisions had a high mortality rate for people aged ≥80 years, especially for other unforeseen accident at 41.39 (95% CI, 41.35–41.43) per 100,000 for males and 12.89 (95% CI, 12.88–12.91) per 100,000 for females, fall at 28.41 (95% CI, 28.38–28.45) per 100,000 males and 11.23 (95% CI, 11.21–11.26) per 100,000 females, and transport accidents at 10.56 (95% CI, 10.42–10.70) per 100,000 males and 3.74 (95% CI, 3.71–3.79) per 100,000 females. Mortality was lowest in the younger age groups (0–4 years and 5–14-years), especially for intentional self-harm in the 0–4 age group at 0.00 per 100,000 for males and females, assault at 0.02 (95% CI, 0.02–0.03) per 100,000 for males and 0.02 (95% CI, 0.02–0.02) per 100,000 for females, and other unforeseen accidents at 0.04 (95% CI, 0.04–0.05) per 100,000 for males and 0.03 (95% CI, 0.03–0.04) per 100,000 for females in the 5–14-year age group.

**Table 2:** The average number of deaths and mortality rates by sex and age group because of intracranial injuries by external cause type from 1999–2021 in Japan.

	External causes of morbidity and mortality		Fall		Assault	
	(V01-Y89)		(W00-W19)		(X85-Y09)	
Age (years)	Average number	Mortality rate (95% CI)	Average number	Mortality rate (95% CI)	Average number	Mortality rate (95% CI)
Male						
0–4	35.0	1.5 (1.47–1.55)	8	0.43 (0.41–0.47)	2.2	0.13 (0.11–0.15)
5–14	43.0	0.81 (0.80–0.83)	4	0.09 (0.09–0.11)	0.4	0.02 (0.02–0.03)
15–44	827.6	3.75 (3.74–3.77)	99.5	0.43 (0.44–0.44)	12.5	0.07 (0.07–0.08)
45–64	1118.1	6.67 (6.65–6.69)	409.4	2.41 (2.40–2.42)	12.5	0.09 (0.09–0.10)
65–79	1954.8	19.68 (19.65–19.72)	832.7	8.33 (8.32–8.35)	8.0	0.09 (0.09–0.10)
≥80	2333.2	79.86 (79.82–79.91)	827.3	28.41 (28.38–28.45)	1.7	0.11 (0.09–0.14)
Overall	6320.5	9.77 (9.78–9.78)	2181.8	3.38 (3.38–3.39)	37.3	0.07 (0.07–0.08)
Female						
0–4	22.7	0.99 (0.97–1.03)	4.1	0.25 (0.23–0.28)	1.4	0.08 (0.07–0.09)
5–14	23.1	0.49 (0.48–0.52)	1.9	0.05 (0.05–0.06)	0.2	0.02 (0.02–0.02)
15–44	277.5	1.26 (1.26–1.28)	26.6	0.12 (0.12–0.13)	3.5	0.02 (0.02–0.02)
45–64	363.3	2.19 (2.18–2.21)	80.7	0.47 (0.47–0.48)	4.0	0.03 (0.03–0.04)
65–79	845.9	7.63 (7.60–7.66)	276.9	2.43 (2.42–2.45)	4.4	0.05 (0.05–0.06)
≥80	1574.4	28.18 (28.15–28.21)	619.5	11.23 (11.21–11.26)	2.6	0.06 (0.06–0.07)
Overall	3108.8	4.57 (4.57–4.57)	1009.7	1.49 (1.49–1.50)	16.1	0.03 (0.03–0.03)
	Intentional self-harm		Transport accident		Other unforeseen accidents	
	(X60-X84)		(V01-V99)		(W00-X59)	
Age (years)	Average number	Mortality rate (95% CI)	Average number	Mortality rate (95% CI)	Average number	Mortality rate (95% CI)
Male						
0–4	0.0	0 (0.00–0.00)	16.3	0.75 (0.73–0.79)	3.1	0.17 (0.15–0.21)
5–14	2.8	0.08 (0.07–0.10)	33.2	0.68 (0.67–0.70)	1.6	0.04 (0.04–0.05)
15–44	172.2	0.71 (0.71–0.71)	473	2.4 (2.39–2.43)	40.2	0.17 (0.17–0.18)
45–64	115	0.68 (0.68–0.69)	360.5	2.42 (2.40–2.44)	160.2	0.92 (0.92–0.93)
65–79	51.5	0.52 (0.52–0.53)	434.2	5.31 (5.26–5.36)	568.4	5.57 (5.57–5.58)
≥80	15.5	0.64 (0.62–0.67)	242.8	10.56 (10.42–10.70)	1206.5	41.39 (41.36–41.43)
Overall	361.1	0.57 (0.58–0.58)	1561.9	2.83 (2.82–2.85)	1980.8	3.24 (3.23–3.25)
Female						
0–4	0.00	0 (0.00–0.00)	11.2	0.53 (0.50–0.56)	2.6	0.14 (0.13–0.16)
5–14	2.3	0.07 (0.06–0.09)	16.6	0.41 (0.40–0.44)	0.2	0.03 (0.03–0.04)
15–44	112.1	0.48 (0.49–0.49)	115.5	0.64 (0.63–0.65)	7.5	0.04 (0.04–0.04)
45–64	67.2	0.38 (0.38–0.39)	156.1	1.13 (1.12–1.15)	4.0	0.24 (0.24–0.24)
65–79	31.6	0.28 (0.28–0.29)	322.6	3.42 (3.39–3.46)	186.9	1.59 (1.59–1.61)
≥80	10.5	0.21 (0.21–0.22)	184.2	3.74 (3.71–3.79)	724.3	12.89 (12.89–12.91)
Overall	224.9	0.34 (0.34–0.34)	806.4	1.38 (1.37–1.39)	963.1	1.47 (1.47–1.48)

The mortality rate is the average number per 100,000 population. CI is the confidence interval.

**Fig 2:**
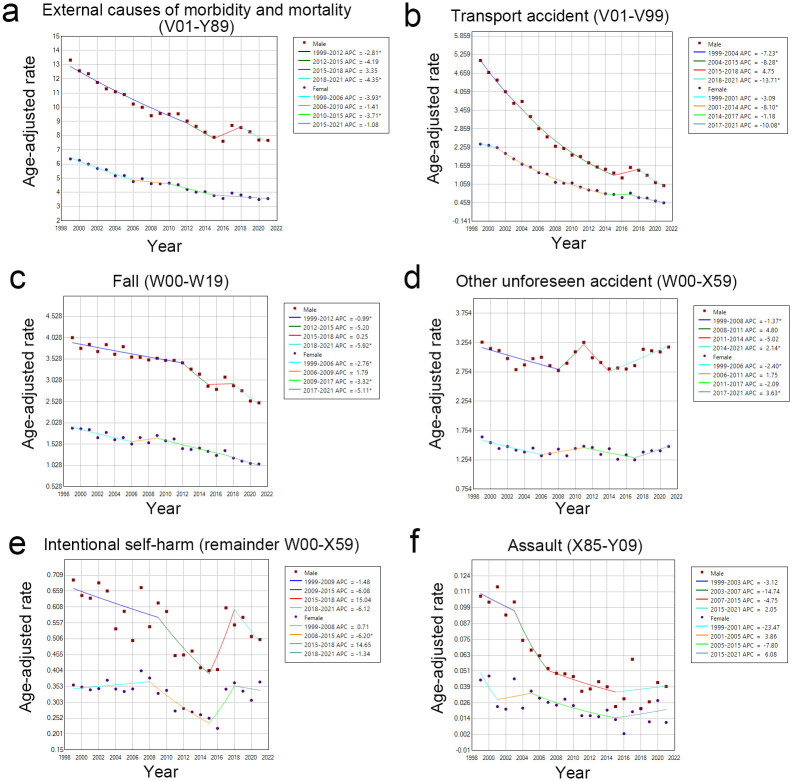
Trends in the age-adjusted rate of change in intracranial injury deaths by external cause type from 1999–2021 in Japan: Analyzed using the JoinPoint regression program for males and females. APC: Annual percentage of change.

The trend in the age-adjusted mortality rate from intracranial injuries by external cause from 1999–2021 was also analyzed using JoinPoint. Both male and female age-adjusted rates decreased in V01–Y89. There was a significant difference in the AAPC of males and females. There was a significant difference in APC from 1999–2012 and 2018–2021 for males, and from 1999–2006 and 2010–2015 for females (Fig 2, S3 and S4 Tables). In the subdivisions of external causes of V01–Y89, the age-adjusted rates from 1999–2021 showed the following: a decreasing trend for transport accidents and falls; flat trend for the other unforeseen accidents except for an increase in 2011; decreasing trend for assault, but with an increase in 2017 for males and in 2018 for females; decreasing trend for intentional self-harm from 1999–2015, but an increasing trend from 2016 to 2021 for males, and a slight increasing trend from 1999–2008, a decreasing trend from 2009 to 2016 and an increase again from 2017 to 2021 for females. There was a significant difference in the AAPC between males and females in transport accidents and falls. There were significant differences in the trend period of the APC: for transport accidents in periods 1999–2004, 2004–2015, and 2018–2021 for males and periods 2001–2014 and 2017–2021 for females; for fall in periods 1999–2012 and 2018–2021 for males and periods 1999–2006, 2009–2017 and 2017–2021 for females. In addition, the APC trend periods with significant differences were the 1999–2008 and 2014–2021 periods for males, and the 1999–2006 and 2017–2021 periods for females in other unforeseen accidents; the 2008–2015 period for females in intentional self-harm; there was no significant difference in assault for both sexes in all periods (Fig 2, S3 and S4 Tables).

### Correlation of mortality between intracranial injuries due to injury type and external cause

In the overall age group, significant inverse correlations in mortality rates for both sexes were observed between falls, and S06.2 and S06.3, except S06.3 for females; between assault, intentional self-harm and transport accident, and S06.5 and S06.6; and between other unforeseen accidents, and S06, S06.2 and S06.3, except S06 for males (Table 3).

**Table 3:** Correlation coefficient and p-value for intracranial injury mortality with significant inverse correlation between external cause type and injury type in Japan.

Mortality from external cause	vs. Mortality from intracranial injury	Spearman rank correlation coefficient (ρ)	*p*-value (Prob>|ρ|)	Inverse correlation and significant
**Sex = Female, Age = ≥80**				
Mortality in fall	Mortality in S06.6	-0.671	0.0005	*
Mortality in transport accident	Mortality in S06.6	-0.7352	0.00001	*
**Sex = Female, Age = Overall**				
Mortality in fall	Mortality in S06.2	-0.497	0.0158	*
Mortality in fall	Mortality in S06.3	-0.2806	0.1946	
Mortality in assault	Mortality in S06.5	-0.7216	0.0001	*
Mortality in assault	Mortality in S06.6	-0.603	0.0023	*
Mortality in intentional self-harm	Mortality in S06.5	-0.6622	0.0006	*
Mortality in intentional self-harm	Mortality in S06.6	-0.4293	0.0409	*
Mortality in transport accident	Mortality in S06.5	-0.8241	0.00001	*
Mortality in transport accident	Mortality in S06.6	-0.9261	0.00001	*
Mortality in other unforeseen accidents	Mortality in S06	-0.6101	0.002	*
Mortality in other unforeseen accidents	Mortality in S06.2	-0.8947	0.00001	*
Mortality in other unforeseen accidents	Mortality in S06.3	-0.7598	0.00001	*
**Sex = Male, Age = 5–14**				
Mortality in intentional self-harm	Mortality in S06	-0.4221	0.0448	*
Mortality in intentional self-harm	Mortality in S06.2	-0.4705	0.0235	*
**Sex = Male, Age = 65–79**				
Mortality in fall	Mortality in S06.6	-0.5104	0.0128	*
Mortality in assault	Mortality in S06.6	-0.6113	0.0019	*
Mortality in transport accident	Mortality in S06.6	-0.6766	0.0004	*
**Sex = Male, Age = ≥80**				
Mortality in intentional self-harm	Mortality in S06.6	-0.6523	0.0007	*
Mortality in transport accident	Mortality in S06.6	-0.8984	0.00001	*
**Sex = Male, Age = Overall**				
Mortality in fall	Mortality in S06.2	-0.6383	0.001	*
Mortality in fall	Mortality in S06.3	-0.5876	0.0032	*
Mortality in assault	Mortality in S06.5	-0.8777	0.00001	*
Mortality in assault	Mortality in S06.6	-0.8229	0.00001	*
Mortality in intentional self-harm	Mortality in S06.5	-0.6864	0.0003	*
Mortality in intentional self-harm	Mortality in S06.6	-0.6047	0.0022	*
Mortality in transport accident	Mortality in S06.5	-0.935	0.00001	*
Mortality in transport accident	Mortality in S06.6	-0.9396	0.00001	*
Mortality in other unforeseen accidents	Mortality in S06	-0.046	0.835	
Mortality in other unforeseen accidents	Mortality in S06.2	-0.8722	0.00001	*
Mortality in other unforeseen accidents	Mortality in S06.3	-0.5856	0.0033	*

Spearman rank correlation of non-parametric statistical test analysis was performed using JMP software. S06: intracranial injury, S06.2: diffuse brain injury, S06.3: focal brain injury, S06.5: traumatic subdural hemorrhage, S06.6: traumatic subarachnoid hemorrhage.

In the ≥80-year age group, significant inverse correlations in mortality rates were found between transport accidents and S06.6 for both sexes; between falls and S06.6 for females; between intentional self-harm and S06.6 for males.

In addition, significant inverse correlations were found in male mortality rates between intentional self-harm, and S06 and S06.2 in the 5–14-year age group and between fall, assault and transport accident, and S06.6 in the 65–79-year age group.

## Discussion

This study showed higher mortality rates for injury types and external causes of intracranial injury in people aged 65 years and older. Conversely, lower mortality rates were observed in people aged 14 years and younger.

The age distribution of patients with TBI is rapidly changing in Japan. Previous reports from the Japan Neurotrauma Data Bank (JNTDB) have shown a decrease in the proportion of TBI in younger persons and an increase in older persons as a proportion of the total population [14, 15]. Older people are more likely to experience TBI because of falls or traffic collisions, which can damage the brain and blood vessels in the skull [16]. Brain atrophy causes it to shrink with age, creating more space in the skull. Consequently, the bridging veins that connect the brain to the skull become more stretched, making them more susceptible to rupture even after minor trauma. This situation leads to intracranial hematomas, including subdural hematomas [17–19]. Older patients are more likely to be taking anticoagulant and antiplatelet medications, which increase the risk of bleeding and make hemostasis more difficult. These medications may delay clotting or increase bleeding and hematoma formation after head trauma [16]. Older patients have reduced intracranial compliance, meaning the skull cannot respond well to changes in intracranial pressure. Increased intracranial pressure due to hemorrhage or brain injury can lead to brain herniation and other life-threatening complications [20].

The reasons for the lower mortality rate in children are as follows: first, the absolute number of TBI is lower in children. According to triennial E-Stat data from 1999–2020, the number of patients undergoing a medical examination for intracranial injuries in the 0–14-year age group was 1.9% of the total number of patients undergoing medical examinations in all age groups (S5 Table) [21]. Second, several anatomical and physiological differences between children and adults may contribute to the lower incidence of intracranial injuries in children. For example, the skulls of children change significantly with age. Open sutures and frontal windows can somewhat buffer intracranial pressure because the fontanelles and sutures close at different times, especially if the increase in intracranial volume is gradual [22]. In addition, the intracranial structures of children have greater brain capacity than adults [23]. It is believed that mechanical loading, acceleration, deformation, and strain on the skull and brain during head impact are reduced [24], resulting in less damage to the brain. Precautions taken to prevent head injuries, such as preventing falls at home and school and promoting the wearing of helmets when riding bicycles, may also contribute to a reduction in deaths because of head injuries among children.

In 2011, there was a particular increase in epidural hemorrhage (S06.4), traumatic subdural hemorrhage (S06.5), traumatic subarachnoid hemorrhage (S06.6), and other unforeseen accidents, including earthquakes. On March 11, 2011, the Great East Japan Earthquake struck, killing approximately 15,000 people and leaving approximately 7,500 missing [25]. The average mortality for intracranial injuries due to other unforeseen accidents estimated for the three prefectures of Iwate, Miyagi, and Fukushima affected by the Great East Japan Earthquake was 39.9 per 100,000 (may be lower), while the average mortality for other regions not affected by the earthquake was 0.71 per 100,000 (may be higher) (S6 Table) [26]. Earthquake-related traumatic brain injury ranked third among the patients with earthquake-related trauma (16.6%). Lacerations and contusions (59.1%) were the most common, while epidural hematomas (9.5%) were the most common intracranial hemorrhages, followed by intracerebral hematomas (7.0%) and subdural hematomas (6.8%). The mortality rate was 5.6% [27]. Head trauma accounted for 25.7% of earthquake-related injuries during the 2008 Wenchuan (Sichuan Province) earthquake in China, with 15% being intracranial injuries [28]. Another hospital reported 17.2% of intracranial injuries from head trauma, the most common being traumatic subarachnoid hemorrhage, and the most common sites were temporal and frontal [29]. The present results support these data. This study did not detect increased diffuse brain injury (S06.2) and focal brain injury (S06.3) mortality in 2011. These findings indicate that S06.2 and S06.3 were not the main causes of earthquake death. The main mechanism of S06.2 is rapid acceleration and deceleration of the head, as seen in high-speed automobile accidents [30]. S06.3 is usually caused by a direct blow to the head [31] and is common in fall [30]. However, most earthquake-induced brain injuries are caused by large and heavy roofs, ceilings, walls of houses, and furniture falling or being drifted by a tsunami. The head does not move so fast and is not in a fall-like situation, which may explain why S06.2 and S06.3 are less common. Data analysis of earthquake disasters should be used in future disaster medicine.

Some injury types were inversely correlated with external causes of intracranial injuries. Significant inverse correlations in male mortality rates were found for intentional self-harm with intracranial injury (S06) and S06.2 in the 5–14-year age group and with S06.6 in the ≥80-year age group. In Japan, hanging from a rope or other cord is the most common method of intentional self-harm in both sexes. In 2003, the proportion of deaths using this method by sex and age group (10-year age group) was the highest for both sexes in all age groups, with “hanging by the neck” accounting for >70% of deaths among males aged ≥60 years and females aged ≥70 years [32]. This lack of intracranial injury resulting from hanging may explain why intentional self-harm was inversely correlated with intracranial injury.

There was a significant inverse association between transport accidents and mortality from S06.5 and S06.6 at all ages for both sexes, especially for males aged 65–79 and over 80 years. In Taiwan, adults over 60 years tend to have higher rates of S06.5 and S06.6 in intracranial injuries due to collision accidents [33].

Mortality caused by falls was significantly inversely correlated with mortality from S06.2 in both sexes and S06.3 in males in all age groups and with mortality from S06.6 in males in the 65–79 and over 80 age groups. In an American study on the characteristics of blunt traumatic supraventricular cranial bleeding (STCB) types, traumatic subdural hemorrhage (S06.5) was the most common STCB (53%), occurring predominantly in older patients (78%) after a fall, with 30% requiring craniotomy and 7% mortality. S06.6 occurred in 32% of the patients and had the lowest mortality rate (3%) [34]. It is indicated that the Japanese mortality data for S06.5 and S06.6 are fairly consistent with the US mortality data for similar conditions. However, in Turkey, S06.6 had the highest mortality rate for head injuries caused by falls, followed by S06.5 [35].

Mortality from assault was significantly inversely correlated with mortality from S06.5 and S06.6 in both sexes in all age groups and with mortality from S06.6 in males in the 65–79 age group. The global incidence of fatal head trauma because of assault is higher than that of nonfatal cases. Most fatal cases involve the use of blunt weapons and firearms. In addition, they were almost always associated with S06.5 or S06.6 [36]. However, the possession of firearms is strictly regulated by law in Japan [37]. This regulation may have resulted in fewer S06.5 and S06.6 due to assault.

Mortality from intentional self-harm was significantly inversely correlated with mortality from S06.5 and S06.6 in both sexes in all age groups, especially in males over 80 years old, and with mortality from S06 and S06.2 in males in the 5–14-year age group. According to a 2012 report by Large, intracranial self-stabbing was associated with a diagnosis of psychotic illness in approximately half of the 49 cases over 10 years. Intracranial self-stabbing is not always performed with suicidal intent and usually does not have a fatal outcome [38]. This might explain the low mortality rate associated with intracranial injuries caused by intentional self-harm.

Although this paper reported statistical analysis of intracranial injury data only for Japan, traumatic brain injury is likely to vary widely depending on the economic situation in each country. Incidence rates vary by region. Regarding the causes of occurrence, for example, the proportion of TBIs resulting from road traffic collisions is greatest in Africa and Southeast Asia (56%) and lowest in North America (25%). Regarding socioeconomic factors, low- and middle-income countries have nearly three times higher rates of TBI than high-income countries [39]. While considering these circumstances, it is necessary to refer to the data analyzed in this paper.

## Conclusion

We characterized the data through statistical analysis of intracranial injury data published by the Japanese government. From 1999–2021, there was a decreasing trend in mortality from intracranial injuries; a higher proportion of deaths from intracranial injuries occurred in persons aged 65 years and older than in younger persons. Intracranial injury types had higher mortality rates due to diffuse brain injury and traumatic subdural hemorrhage. Among the external causes of intracranial injury, mortality from falls, transport accidents, and other unforeseen accidents was high. For some age groups and sexes, there were significant inverse correlations of mortality with traumatic subdural hemorrhage and traumatic subarachnoid hemorrhage for transport accidents, intentional self-harm and assault, and diffuse brain injury and focal brain injury for falls. Prevention of falls in the elderly and reduction of the external forces on the head during falls is important to reduce the number of deaths because of head trauma. We believe that the data presented in this analysis will be useful for preventing and treating intracranial injuries and developing administrative measures to reduce intracranial injuries. In particular, this paper showed an inverse correlation between the type of intracranial injury and the type of external cause. We hope that this will contribute to the ability of clinicians to infer the type of intracranial injury from external factors in dying patients with intracranial injuries and to initiate treatment as early as possible before a thorough examination.

## Supporting information

S1 Table Trends in the statistical rate of change in intracranial injury deaths by injury type in 1999–2021 were analyzed using the JoinPoint regression program for males in Japan. APC: Annual percentage change, AAPC: Average annual percentage change, CI: confidence interval.
(XLSX)

S2 Table Trends in the statistical rate of change in intracranial injury deaths by injury type in 1999–2021 were analyzed using the JoinPoint regression program for females in Japan. APC: Annual percentage change, AAPC: Average annual percentage change, CI: confidence interval.
(XLSX)

S3 Table Trends in the statistical rate of change in intracranial injury deaths by external cause type in 1999–2021 were analyzed using the JoinPoint regression program for males in Japan. APC: Annual percentage change, AAPC: Average annual percentage change, CI: confidence interval.
(XLSX)

S4 Table Trends in the statistical rate of change in intracranial injury deaths by external cause type in 1999–2021 were analyzed using the JoinPoint regression program for females in Japan. APC: Annual percentage change, AAPC: Average annual percentage change, CI: confidence interval.
(XLSX)

S5 Table Number of intracranial injuries and deaths due to intracranial injuries at 3-year intervals in 1999–2020 in Japan. (DOCX)

S6 Table Estimated mortality per 100,000 persons from intracranial injuries due to other unforeseen accidents in the earthquake-affected regions, earthquake-free regions, and on a national level. (XLSX)
