# Supplementary material for: Trends in mortality rates and correlations between intracranial injuries and external causes: A Japanese population study
Source: PLoS One. 2024 May 8;19(5):e0300846. doi: 10.1371/journal.pone.0300846 (PMC11078349; doi:10.1371/journal.pone.0300846)
Supplement: S5 Table — (DOCX) [file pone.0300846.s005.docx]

S5 Table. Number of intracranial injuries and deaths due to intracranial injuries at 3-year intervals in 1999–2020 in Japan.

| Number of intracranial injuries | | | |  |  |  |  |
| --- | --- | --- | --- | --- | --- | --- | --- |
| Year | Overall | 0–4 | 5–14 | 15–44 | 45–64 | 65–79 | 80– |
| 1999 | 11,200 | 200 | 200 | 1,500 | 2,600 | 3,900 | 2,900 |
| 2002 | 11,500 | 100 | 200 | 1,400 | 2,300 | 4,200 | 3,400 |
| 2005 | 13,000 | 100 | 200 | 1,200 | 2,300 | 4,800 | 4,300 |
| 2008 | 13,800 | 100 | 200 | 1,200 | 2,200 | 4,900 | 5,200 |
| 2011 | 14,700 | 0 | 200 | 1,000 | 2,100 | 5,300 | 6,100 |
| 2014 | 13,700 | 0 | 200 | 1,000 | 1,800 | 4,600 | 5,600 |
| 2017 | 16,800 | 100 | 100 | 9,00 | 2,000 | 5,300 | 8,500 |
| 2020 | 15,800 | 100 | 100 | 7,00 | 2,000 | 4,600 | 8,100 |
| Average | 13,813 | 88 | 175 | 1,113 | 2,163 | 4,700 | 5,513 |
| Number of deaths due to intracranial injuries | | | | |  |  |  |
| 1999 | 9,791 | 103 | 123 | 1,887 | 2,194 | 2,939 | 2,517 |
| 2002 | 9,301 | 88 | 106 | 1,612 | 1,998 | 2,926 | 2,556 |
| 2005 | 9,097 | 70 | 96 | 1,329 | 1,715 | 2,935 | 2,941 |
| 2008 | 8,522 | 53 | 60 | 990 | 1,419 | 2,717 | 3,280 |
| 2011 | 9,113 | 39 | 58 | 872 | 1,324 | 2,691 | 4,119 |
| 2014 | 8,637 | 39 | 42 | 696 | 1,057 | 2,519 | 4,279 |
| 2017 | 9,494 | 36 | 31 | 774 | 1,148 | 2,620 | 4,880 |
| 2020 | 8,830 | 29 | 31 | 617 | 932 | 2,251 | 4,970 |
| Average | 9,098 | 57 | 68 | 1,097 | 1,473 | 2,700 | 3,693 |
